# Conventional and biologic disease-modifying anti-rheumatic drugs for osteoarthritis: a meta-analysis of randomized controlled trials

**DOI:** 10.1093/rheumatology/key131

**Published:** 2018-06-16

**Authors:** Monica S M Persson, Aliya Sarmanova, Michael Doherty, Weiya Zhang

**Affiliations:** Academic Rheumatology, Arthritis Research UK Pain Centre, University of Nottingham, Nottingham, UK

**Keywords:** osteoarthritis, DMARDs, biologics, methotrexate, hydroxychloroquine, inflammation, meta-analysis

## Abstract

**Objectives:**

The role of inflammation in OA is controversial and it is unclear whether suppressing inflammation with conventional or biologic DMARDs is effective. A systematic review and meta-analysis of randomized controlled trials (RCTs) was conducted to compare DMARDs with placebo in participants with symptomatic OA.

**Methods:**

Databases (Medline, Embase, Allied and Complementary Medicine Database, Web of Science and Cochrane Library), conference abstracts and ClinicalTrials.gov were searched to end of November 2017 for placebo-controlled RCTs of DMARDs, including biologics, in symptomatic OA. Pain data at treatment peak time point were extracted and combined using a random-effects meta-analysis. Markers of inflammation and adverse events were extracted and reviewed. Risk of bias assessment was conducted using Cochrane’s tool.

**Results:**

Eleven RCTs (1205 participants) were meta-analysed, including six for conventional DMARDs (757 participants) and five for biologics (448 participants). Overall, DMARDs were statistically superior to placebo [effect size (ES) = 0.18, 95% CI: 0.03, 0.34], although the difference was not clinically significant (0.5 ES threshold). Furthermore, no statistically significant differences were observed in sub-analysis of high-quality trials (ES = 0.11, 95% CI : −0.06, 0.28), biologics (ES = 0.16, 95% CI: −0.02, 0.34) or conventional DMARDs (ES = 0.24, 95% CI: −0.05, 0.54). No difference was found between erosive *vs* non-erosive hand OA, hand *vs* knee OA or anti-IL1 *vs* anti-TNF biologics.

**Conclusion:**

DMARDs did not offer clinically significant pain relief above placebo in OA. This poor efficacy indicates that inflammation may not be a prime driver for OA pain.

**Rheumatology key messages**

- No clinically significant pain relief is offered by DMARDs (compared with placebo) in OA.
- No statistically significant difference was observed between various drugs, drug targets, joint sites, or OA subtypes.
- Inflammation in OA may not be a major risk factor for OA pain.

## Introduction

OA is a major cause of pain and disability for which no disease-modifying drug interventions have yet been identified. It is a common complex disorder that involves all joint tissues and affects approximately 1 in 5 women and 1 in 10 men aged >60 years [1]. The pathogenesis of OA has not been fully characterized, and the role of synovial inflammation within this process is intensely debated.

Some perceive OA as the inherent repair process of synovial joints in which modest inflammation is secondary to joint tissue damage caused largely by biomechanical insult [2]. In contrast, others believe synovial inflammation to be a more primary feature and a central driver of OA pain and progression [3]. This belief has encouraged the conduct of randomized controlled trials (RCTs) of conventional and biologic DMARDs in OA. These potent agents are used in inflammatory arthritides, such as RA, where they suppress the aggressive primary inflammation that drives the disease [4].

Conventional and biologic DMARDs are the two major classes of DMARDs used for RA. Conventional DMARDs include drugs such as MTX and HCQ [4], whereas biologic DMARDs are mAbs and soluble receptors that target protein messenger molecules or cells [4].

Whether DMARDs are effective for OA remains controversial. The literature is scattered with hypotheses and hopes for a positive effect in OA. However, this is interspersed with reports of treatment failures. We therefore undertook the present meta-analysis to examine the efficacy of DMARDs, including both conventional and biologic DMARDs, in participants with symptomatic OA.

## Methods

Placebo-controlled RCTs comparing a DMARD, including biologics, with placebo in participants with symptomatic OA at any site were included. DMARDs that are recommended or licensed for RA were considered for this review [5, 6]. Supplementary Table S1, available at *Rheumatology* online, lists all drugs eligible for inclusion in the review. Full text publications and conference abstracts in any language were accepted. No limits were set for publication year.

A systematic literature search was conducted across five databases: Medline, Embase, Allied and Complementary Medicine Database, Web of Science and Cochrane Library. The full search strategies are available in supplementary Table S2, available at *Rheumatology* online. The search was run from date of database inception to 30 November 2017. Additional trials were searched for using the online clinical trial register ClinicalTrials.gov and EULAR, OARSI and ACR annual meeting abstracts. Following the removal of duplicates, all trials were examined for eligibility. Full texts of eligible abstracts were sought and used. Where full texts were unavailable, conference abstracts were included to minimize publication bias and to ensure the evidence captured was current. The data were extracted independently by two reviewers (M.S.M.P. and A.S.) using a Microsoft Access extraction form created for this review. The following information was extracted: publication details, including author, journal, year and publication type (full text or abstract); trial details, including trial funder, study design, blinding and duration; participant details and demographics, including number of participants, age, gender distributions and BMI; the joint affected; method of diagnosis (e.g. clinical, radiographic); OA subset details; and intervention/control details, including the drug, formulation, dose and frequency.

Pain data at treatment peak time point were extracted. Treatment peak time point (i.e. time point where treatment group had the greatest improvement) was chosen under the assumption that if a difference between treatment and placebo was not observed at this time point, one could confidently assume no efficacy at other time points. Where multiple tools for assessing pain were presented, the outcome was chosen using the hierarchy defined by Jüni *et al*. [7]. Where possible, changes from baseline pain scores were extracted/calculated; if not, end point scores were used to calculate the between-group mean differences. If pain outcomes were dichotomized, the percentage of participants with improvement in pain (as defined in the publication) was used. Data were preferentially extracted from intention-to-treat analyses. Data were not extracted from graphs, and missing data were not sought from investigators. Where a trial examined multiple dosages of the intervention, these were combined into one group for analyses.

Other outcomes extracted were inflammation (local or systemic) and incidence of adverse events (AEs).

### Risk of bias

Two reviewers independently assessed the risk of bias of included trials using Cochrane’s Risk of Bias Tool [8]. The distribution of scores for each domain was presented.

### Statistical methods

Hedges’ effect size (ES) and corresponding 95% CI were calculated. If dichotomized, the odds ratio for improvement was extracted/calculated and converted to an ES [8, 9]. The study estimates were combined using a random-effects meta-analysis weighted using inverse-variance methods. Heterogeneity was quantified using *I*^2^, and the *P*-value was calculated using the *Q* test [10, 11]. Publication bias was presented using a funnel plot, and the asymmetry of this plot was examined by Egger’s test [12]. As recommended in the National Institute for Health and Care Excellence guidelines for OA, a minimal clinically important difference threshold of ES = 0.5 for the lower level of the 95% CI was used to determine clinical significance [13].

All DMARDs were pooled before being examined by type (conventional *vs* biologic). Further subgroup analysis was conducted to examine biologics by the mechanism of action (TNF-inhibitor *vs* IL1-inhibitor). Additional subgroup analyses were conducted by joint affected, OA subtype (erosive *vs* non-erosive hand OA) and publication type. A sensitivity analysis was only conducted for high-quality trials using adequate allocation concealment as an indicator of quality [14].

Analyses were done with Stata (StataCorp. 2015. Stata Statistical Software: Release 14. College Station, TX, USA: StataCorp LP). The trial was registered with PROSPERO (PROSPERO 2017 CRD42017067427).

## Results

### Description of trials

**Fig. 1:**
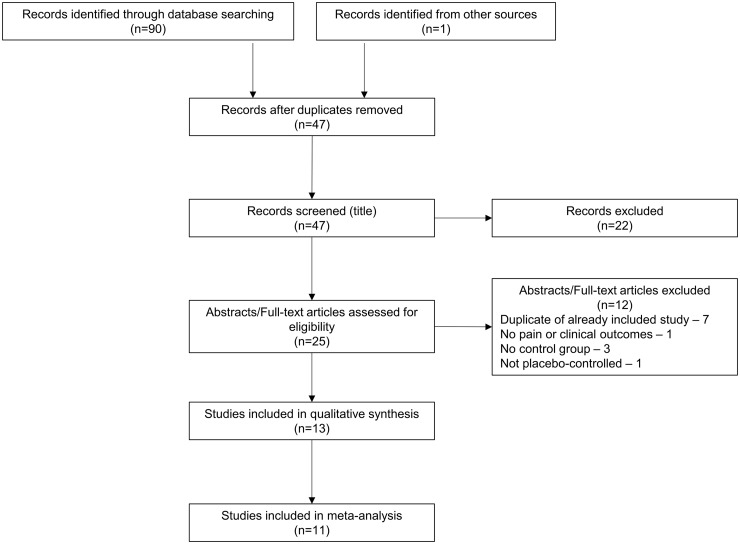
PRISMA flow diagram Results of the literature search.

Thirteen studies were identified comparing a DMARD with placebo in participants with OA (Fig. 1 and Table 1). Outcome data were not available for extraction from two abstracts [15, 16]. The meta-analysis is based on the remaining 11 RCTs (7 full texts, 4 abstracts), including 6 for conventional [25–27] and 5 for biologic [18–20, 24, 23] DMARDs. A variety of DMARDs were examined, including HCQ (5 trials) [17, 21, 22, 26, 27], MTX (1 trial) [25], anakinra (ANK; 1 trial) [19], adalimumab (ADA, 3 trials) [18, 20, 24] and etanercept (ETN; 1 trial) [23]. Ten trials were parallel design trials, while one trial [18] was a cross-over design trial and combined both treatment periods. Median trial duration was 24 weeks (range 12–52 weeks).

**Table 1:** Description of trials comparing DMARD with placebo in OA

References	Funding source	Design	Drug	Route	Dose	Study duration (week)	Duration analysed (week)	No. randomized (n on DMARD)	No. of female (%)	Mean age (s.d.)	Joint	OA detail
Abou-Raya *et al.* [16]	Unclear	Parallel	MTX	Oral	25 mg/week + usual treatment	16	Not analysed	88 (44)	NP	NP	Knee	OA
Abou-Raya *et al.* [17]	Unclear	Parallel	HCQ	Oral	400 mg/day	36	16	166 (83)	NP	NP	Knee	OA + clinical signs of synovitis
Aitken *et al.* [18]	Unclear	Cross-over	ADA	SC	40 mg/every other week	12	12	43 (43)	NP	61 (8.4)	Hand	OA + synovitis (erosive hand OA)
Chevalier *et al.* [19]	Commercial	Parallel	ANK	IA	50 mg or 150 mg (single injection)	12	4	170 (101)	107 (63)	62.6 (9.7)	Knee	OA, no effusion
Chevalier *et al.* [20]	Commercial	Parallel	ADA	SC	40 mg (two injections separated by 2-week interval)	26	6	85 (42)	71 (84)	62.5 (6.9)	Hand	OA refractory to analgesics
Jokar *et al.* [21]	Public	Parallel	HCQ	Oral	400 mg/day	24	21	51 (25)	43 (98)	47.9 (9.8)	Knee	OA
Kingsbury *et al.* [22]	Public	Parallel	HCQ	Oral	Dose dependant on gender, height and weight	52	52	248 (114)	NP	NP	Hand	OA
Kloppenburg *et al.* [23]	Commercial	Parallel	ETA	SC	50 mg/week (first 24 weeks), thereafter 25 mg/week	52	52	90 (45)	NP	60	Hand	OA + synovitis (erosive hand OA)
Lindsley *et al.* [15]	Unclear	Parallel	INF	IA	100 mg (single injection)	8	Not analysed	16 (8)	NP	NP	Knee	OA
Verbruggen *et al.* [24]	Commercial	Parallel	ADA	SC	40 mg/every other week	52	52	60 (30)	NP	61.3 (6.5)	Hand	OA + synovitis (erosive hand OA)
Holanda *et al.* [25]	Unclear	Parallel	MTX	Oral	7.5 mg/week	17	17	58 (29)	48 (83)	61.1 (8.8)	Knee	OA
Bonfante *et al.* [26]	Unclear	Parallel	HCQ	Oral	400 mg/day	16	16	32 (16)	26 (81)	60.7 (9.6)	Knee	OA
Lee *et al.* [27]	Unclear	Parallel	HCQ	Oral	400 mg/day	24	24	196 (98)	168 (86)	58 (7.6)	Hand	OA

ADA: adalimumab; ANK: anakinra; ETA: etanercept; IA: intraarticular; INF: infliximab; NP: not presented.

A total of 1205 participants with clinically and radiographically confirmed OA were included in the meta-analysis. Five trials examined participants with knee OA, of which one [17] was restricted to participants with clinical signs indicative of synovitis. In contrast, the RCT by Chevalier *et al.* [19] excluded participants with effusions and inflammatory flares. The remaining six trials studied participants with hand OA. Three of these trials [18, 23, 24] were restricted to participants with erosive hand OA. In addition, a further hand OA trial was limited to participants with OA that was refractory to analgesics [20].

In the six studies that presented the gender distributions of participants, approximately three-quarters of the participants were women (78.2%). The mean age of participants in the eight trials where age data were available ranged from 47.9 years [21] to 62.6 years [19].

### Bias within and across trials

**Fig. 2:**
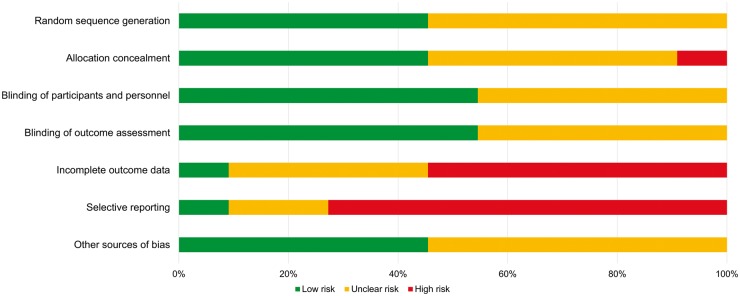
Risk of bias of analysed trials Cochrane’s Risk of Bias assessment [8] across all trials included in the meta-analysis. Percentage of trials scoring low risk, unclear risk and high risk of bias across seven domains of bias.

Potential risk of bias was demonstrated in the analysed trials (Fig. 2). The primary sources of bias were selective outcome reporting and incomplete outcome data, where over half the studies were associated with high risks of bias. Online trial registrations were found for six studies, and a published protocol was found for one study. These were compared with the outcomes available in the included publication, and the publications most commonly did not include all pre-specified outcomes, did not fully present the outcomes or conducted analyses that had not been pre-specified. Furthermore, many trials excluded participants from analysis [17, 18, 22, 23], used inappropriate methods for imputation of data (e.g. last observation carried forward) [19, 24], or did not provide sufficient detail regarding the amount of missing data or how it was handled [20, 21, 25, 26, 27]. These domains cause concerns about the risk of bias of the included trials.

All trials were reported as double-blinded; however, the adequacy of the blinding methods could not be determined in 5 of the 11 trials, due to insufficient information in the publications. The remaining RCTs described appropriate procedures for blinding of participants, physicians and outcome assessors.

Nearly half (45%) of the studies reported adequate methods of allocation concealment, most commonly through central allocation external to the study investigators. One trial [25] allocated treatment by the order of enrolment to the trial, and this was deemed to have a high risk of bias. There was no evidence of funnel plot asymmetry (*P* = 0.121) (supplementary Fig. S1, available at *Rheumatology* online).

### Efficacy of DMARDs in OA

#### Pain

**Fig. 3:**
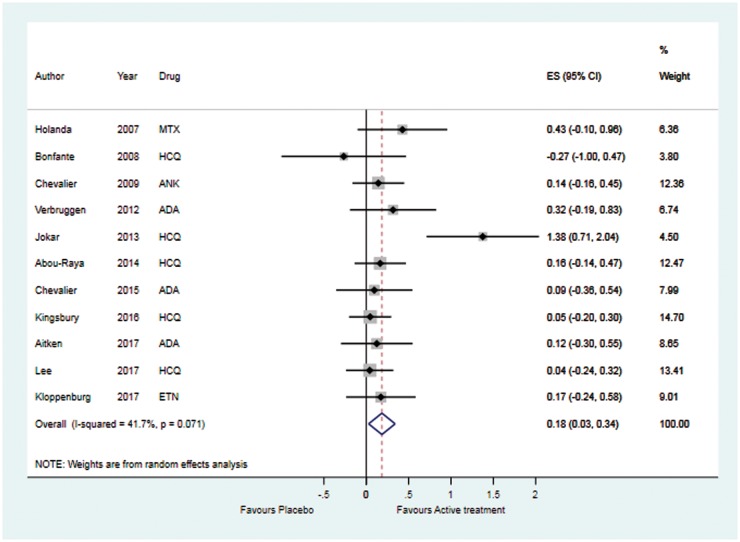
Forest plot of DMARDs *vs* placebo in OA Hedges’ effect size (ES) and 95% CI presented for pain at peak time point for intervention arm. ADA: adalimumab; ANK: anakinra; ETN: etanercept.

Overall, the pooled result of outcomes for conventional and biologic DMARDs was statistically superior to that for outcomes for placebo with respect to pain relief (Fig. 3 and Table 2). The ES was 0.18 (95% CI: 0.03, 0.34). The estimate was associated with a moderate level of inconsistency (*I*^2^ = 41.7%) [8]; however, the test for heterogeneity was not statistically significant (*P* = 0.071). A sensitivity analysis including only high-quality trials, using adequate allocation concealment as a quality indicator, showed no difference between DMARDs and placebo (ES = 0.11, 95% CI: −0.06, 0.28) (Table 2). No heterogeneity was evident in the estimate. Furthermore, separate examination of conventional and biologic DMARDs found that neither conventional (ES = 0.24, 95% CI: −0.05, 0.54) nor biologic (ES = 0.16, 95% CI: −0.02, 0.34) DMARDs were superior to placebo for pain relief. Large and significant heterogeneity was observed across the conventional DMARD trials. However, the results for biologic DMARDs were homogeneous, and this homogeneity was retained when examined by mechanism of action. Neither IL1-inhibitors (ES = 0.14, 95% CI: −0.16, 0.45) nor TNF-inhibitors (ES = 0.17, 95% CI: −0.05, 0.54) were effective.

**Table 2:** Overall, sensitivity and subgroup meta-analysis results for biologic and conventional DMARDS in OA

Analysis	No. of trials	No. analysed	ES (95% CI)	*I*^2^ (*P*-value)
Overall	11	1205	0.18 (0.03 to 0.34)	41.7% (0.071)
Sensitivity analysis				
Allocation concealment	5	765	0.11 (−0.06, 0.28)	0.0% (0.818)
Subgroup analysis				
DMARD type				
Biologic DMARD	5	448	0.16 (−0.02, 0.34)	0.0% (0.975)
IL1-inhibitors	1	170	0.14 (−0.16, 0.45)	.
TNF-inhibitors	4	278	0.17 (−0.05, 0.39)	0.0% (0.926)
Conventional DMARD	6	757	0.24 (−0.05, 0.54)	70.0% (0.005)
Joint				
Knee	5	477	0.34 (−0.05¸0.73)	71.7% (0.007)
Hand	6	728	0.09 (−0.05, 0.24)	0.0% (0.948)
OA type				
Erosive hand OA	3	193	0.19 (−0.06, 0.45)	0.0% (0.846)
Non-erosive hand OA	3	535	0.05 (−0.12, 0.22)	0.0% (0.979)
Publication type				
Conference abstract	4	547	0.11 (−0.05, 0.27)	0.0% (0.925)
Full text	7	658	0.27 (−0.01, 0.55)	62.7% (0.013)

Presented as Hedges’ effect size (ES) and associated 95% CI. *I*^2^ (the variation in ES attributable to heterogeneity) and associated *P*-values are presented.

Further subgroup analyses found that the lack of efficacy of treatment compared with placebo did not vary by the joint site (hand *vs* knee OA), inflammatory phenotype (erosive *vs* non-erosive hand OA) or publication type (conference abstract or full text).

#### Inflammation and AEs

Only six studies assessed inflammation. One found a statistically significant improvement (US synovitis) [16], one found a clinically relevant improvement (clinical and US) [17], and four found no difference between treatment and placebo (MRI, serum and tissue CRP, effusion) [15, 18, 20, 24]. However, no quantitative data were available for further analysis. AE incidence was poorly reported across trials and was not aggregated.

## Discussion

This first meta-analysis of conventional and biologic DMARDs in OA, including 11 RCTs with over 1200 participants, did not demonstrate statistically significant pain relief from either conventional or biologic DMARDs compared with placebo. Biologics, in particular, showed homogeneity across studies. Conventional and biologic DMARDs, when combined, were statistically superior to placebo (ES = 0.18, 95% CI: 0.03, 0.34), but this was far below the minimal clinically important difference threshold (ES = 0.5) used in the UK [13]. Moreover, examination of only high-quality trials with adequate allocation concealment did not produce statistically significant results (ES = 0.11, 95% CI: −0.06, 0.28), and this estimate was homogeneous. Any benefit observed is likely overestimated by the risk of bias associated with selective outcome reporting [8]. Furthermore, the analysis is based on peak time point for the intervention, so even at their most effective time-point these treatments do little better than placebo.

Although the use of biologic DMARDs in OA has been heralded as a promising way forward based on *in vitro*, animal model and uncontrolled pilot studies, narrative reviews have indicated that they do not appear to have any clear benefit over placebo in RCTs [28–30]. This is confirmed by the present meta-analysis, which is able to provide quantitative evidence for a lack of statistical superiority of biological DMARDs over placebo. In contrast, the perceived efficacy of conventional DMARDs in the literature is mixed. For example, the ACR specifically advises against the use of MTX and does not offer any recommendations on HCQ due to limited evidence [31], whereas others favour a positive effect for MTX and HCQ [30, 32]. However, the previously reviewed evidence is limited to retrospective or non-blinded trials [32] and the results of an RCT for MTX that has since been retracted due to serious flaws and concerns about the reliability of the data [33].

Targeting the innate immune response (through IL1), adaptive immune response (through TNF) and the overall level of inflammation (through MTX) without major effects on pain suggests that inflammation may not be a key driver of OA pain [2]. This view is further supported by the lack of efficacy across the spectrum of inflammatory phenotypes (from no effusion/inflammatory flares to synovitis or erosive hand OA) and joint sites. The poor efficacy of these drugs, which are used for RA [5], may indicate that the quantity and/or role of inflammation in OA differs from the primary aggressive inflammation in inflammatory arthritides. Alternatively, the investigated drugs may not be targeting the correct inflammatory pathways or may not be using the drug at sufficient exposures to capture a clinical effect. Better understanding of pain mechanisms and the role of inflammation in OA is required in order to identify and develop more effective treatments.

### Limitations

The study is subject to several limitations. First, the meta-analysis included conference abstracts that have not been subject to the more stringent level of peer review as full texts. Furthermore, abstracts do not allow full examination of methods and critical appraisal of risks of bias. As a result, the overall risk of bias of the included trials was difficult to ascertain. However, the inclusion of abstracts ensured that the evidence captured was as complete as possible, which was partially reflected by the lack of publication bias [34]. Second, data were not sought from investigators and two trials were not included in the meta-analysis as pain data were not available for extraction from the conference abstracts. However, those trials included a total of 90 participants and their exclusion represents a loss of only 7% of the total participant population. Third, it was not possible to examine inflammation and AEs in a meta-analysis due to lack of information and inconsistent reporting. Fourth, the overall results and conventional DMARD-specific results were associated with considerable and significant heterogeneity. The predominant source of heterogeneity was the RCT by Jokar *et al.* [19], which had a considerably younger [mean (s.d.) age 47.9 (9.8)] and predominantly (98%) female population with overall low degrees of radiographic OA. This trial reported an extraordinarily high ES for HCQ (1.38, 95% CI: 0.71, 2.04). Its exclusion from analysis increased homogeneity (to *I*^2^= 0.0%); however, the conclusions remained unchanged. Fifth, subgroup analyses were based on relatively small numbers of trials, which may limit the power of this meta-analysis to detect significant differences within the subgroups. Finally, the use of treatment peak time point as the time point for analyses likely overinflates the effect size estimate. However, it was chosen under the assumption that if no difference was evident at the most effective time-point, then the conventional and biologic DMARDs were unlikely to be effective. It also allowed more trials to be analysed. Displaying only a small effect at their peak, conventional and biologic DMARDs are unlikely to have considerable, or in fact any, effect at other time points.

## Conclusion

Neither conventional nor biological DMARDs relieve pain due to OA. There is no difference between anti-IL1 and anti-TNF biologics, and no difference between treating erosive *vs* non-erosive hand OA or hand *vs* knee OA. Although combining all DMARDs provided statistically significant pain relief, this was not clinically significant, nor was it supported by sensitivity analyses of high-quality trials. The results suggest that inflammation may not be a principal risk factor for OA pain.

*Funding*: This work was supported by Arthritis Research UK [grant number 20777].

*Disclosure statement*: M.D. reports a grant from AstraZeneca funding a non-drug PI-led study in Nottingham (Sons of Gout Study) and honoraria for Advisory Boards on osteoarthritis and gout for AstraZeneca, Grunenthal, Mallinckrodt and Roche, outside the submitted work. W.Z. reports honoraria for Grunenthal and speaker fees for Bioiberica and Hisun, outside the submitted work. All other authors have declared no conflicts of interest.

## Supplementary Material

Supplementary Data
