# Supplementary material for: Conventional and biologic disease-modifying anti-rheumatic drugs for osteoarthritis: a meta-analysis of randomized controlled trials
Source: Rheumatology (Oxford). 2018 Jun 16;57(10):1830–7. doi: 10.1093/rheumatology/key131 (PMC6199417; doi:10.1093/rheumatology/key131)
Supplement: Supplementary Data [file key131_suppl_data.docx]

**SUPPLEMENTARY DATA**

**Supplementary Table S1. DMARDs eligible for inclusion to the review**

| DMARD type | Drugs |
| --- | --- |
| Conventional synthetics | Methotrexate  Leflunomide  Sulfasalazine  Hydroxychloroquine |
| Targeted synthetics | Tofacitibin  Baricitinib |
| Biologics | Adalimumab  Certolizumab pegol  Etanercept  Golimumab  Infliximab  Abatacept  Rituximab  Tocilizumab  Clazakizumab  Sarilumab  Sirukumab  Anakinra |
| Biosimilars | Amgevita  Benepali  Blitzima  Cyltezo  Erelzi  Flixabi  Imraldi  Inflectra  Remsima |

**Supplementary Table S2. Search strategy implemented**

| Database | Search Strategy |
| --- | --- |
| Medline/Embase/AMED via Ovid | 1. Osteoarthritis.ti.  2. (Methotrexate or leflunomide or sulfasalazine or hydroxychloroquine or adalimumab or certolizumab or etanercept or golimumab or infliximab or abatacept or rituximab or tocilizumab or clazakizumab or sarilumab or sirukumab or tofacitinib or baricitinib or amgevita or benepali or blitzima or cyltezo or erelzi or flixabi or imraldi or inflectra or remsima or anakinra).ab.  3. Randomized controlled trial (MESH)  4. 1 and 2 and 3 |
| Cochrane | 1. osteoarthritis.ti  2. Methotrexate or leflunomide or sulfasalazine or hydroxychloroquine or adalimumab or certolizumab or etanercept or golimumab or infliximab or abatacept or rituximab or tocilizumab or clazakizumab or sarilumab or sirukumab or tofacitinib or baricitinib or amgevita or benepali or blitzima or cyltezo or erelzi or flixabi or imraldi or inflectra or remsima or anakinra  3. #1 and #2  4. Limit #3 to Trials |
| Web of Science | 1. Title: Osteoarthritis  2. Topic: Methotrexate or leflunomide or sulfasalazine or hydroxychloroquine or adalimumab or certolizumab or etanercept or golimumab or infliximab or abatacept or rituximab or tocilizumab or clazakizumab or sarilumab or sirukumab or tofacitinib or baricitinib or amgevita or benepali or blitzima or cyltezo or erelzi or flixabi or imraldi or inflectra or remsima or anakinra  3. Topic: Randomi* controlled trial  4. 1 and 2 and 3 |

**Supplementary Figure S1. Conventional and biologic DMARDs for pain relief in OA**


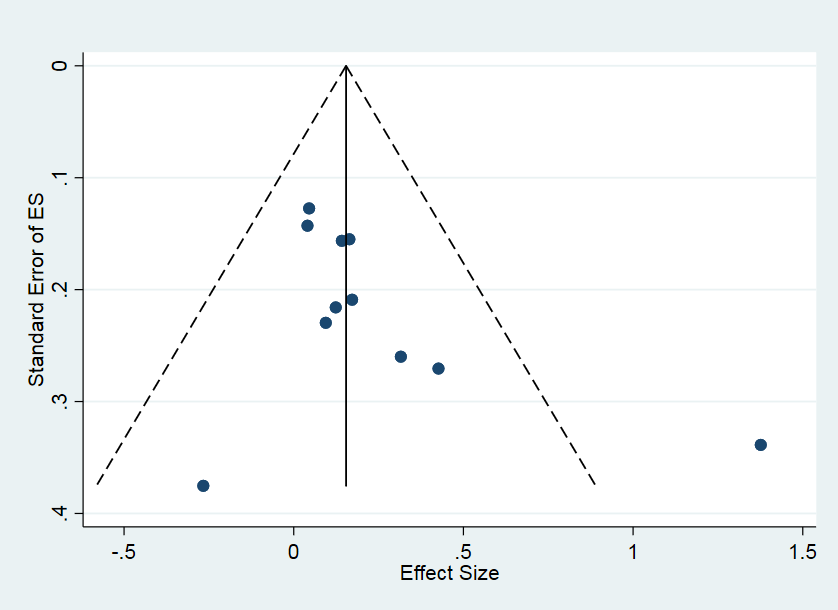


Funnel plot with pseudo 95% confidence limits. Effect size (ES) and standard error of ES of all trials included in the meta-analysis.
